# The Role of the Renin-Angiotensin System and Vitamin D in Keloid Disorder—A Review

**DOI:** 10.3389/fsurg.2019.00067

**Published:** 2019-11-26

**Authors:** Ethan J. Kilmister, Claudia Paterson, Helen D. Brasch, Paul F. Davis, Swee T. Tan

**Affiliations:** ^1^Gillies McIndoe Research Institute, Wellington, New Zealand; ^2^Wellington Regional Plastic, Maxillofacial & Burns Unit, Hutt Hospital, Wellington, New Zealand

**Keywords:** keloid disorder, keloid lesion, renin-angiotensin system, embryonic stem cells, vitamin D deficiency, keloid-associated lymphoid tissue, cathepsins, immune system

## Abstract

Keloid disorder (KD) is a fibroproliferative condition characterized by excessive dermal collagen deposition in response to wounding and/or inflammation of the skin. Despite intensive research, treatment for KD remains empirical and unsatisfactory. Activation of the renin-angiotensin system (RAS) leads to fibrosis in various organs through its direct effect and the resultant hypertension, and activation of the immune system. The observation of an increased incidence of KD in dark-skinned individuals who are predisposed to vitamin D deficiency (VDD) and hypertension, and the association of KD with hypertension and VDD, all of which are associated with an elevated activity of the RAS, provides clues to the pathogenesis of KD. There is increasing evidence implicating embryonic-like stem (ESC) cells that express ESC markers within keloid-associated lymphoid tissues (KALTs) in keloid lesions. These primitive cells express components of the RAS, cathepsins B, D, and G that constitute bypass loops of the RAS, and vitamin D receptor (VDR). This suggests that the RAS directly, and through signaling pathways that converge on the RAS, including VDR-mediated mechanisms and the immune system, may play a critical role in regulating the primitive population within the KALTs. This review discusses the role of the RAS, its relationship with hypertension, vitamin D, VDR, VDD, and the immune system that provide a microenvironmental *niche* in regulating the ESC-like cells within the KALTs. These ESC-like cells may be a novel therapeutic target for the treatment of this enigmatic and challenging condition, by modulating the RAS using inhibitors of the RAS and its bypass loops and convergent signaling pathways.

## Introduction

Keloid disorder (KD) is a fibroproliferative condition characterized by excessive dermal collagen deposition in response to wounding such as ear piercing, surgery and burns, or inflammation such as acne and herpes zoster infection (1–3). Keloid lesions (KLs) characteristically extend beyond the confines of the original wound and invade adjacent dermis without spontaneous scar remodeling typically seen in normal wound healing (2). KLs differ from hypertrophic scars, which are confined within the original wound boundaries and undergo spontaneous regression over time (4).

Keloid lesions present as firm, rubbery, and shiny plaque-like lesions that cause cosmetic concerns, pruritis (5), tenderness (6), and functional problems such as joint contractures (5). *Acne keloidalis nuchae*, a chronic inflammatory condition characterized by scarring around hair follicles, is often complicated by keloid-like papules (5, 7). KLs, especially larger lesions, may cause physical disability and psychological sequelae with impaired quality of life for affected individuals (6, 8).

Keloid lesions occur most commonly on the shoulders, anterior chest, upper back, and earlobes (9, 10). Surface tension and increased sebaceous gland density have been proposed as contributing factors to keloid formation that commonly affect these sites (11), although these do not fully explain earlobes as a site of predilection. KD has a common age of onset between the first and third decades of life, affects both sexes equally and is more common in dark-skinned individuals (10, 12), often displaying a familial pattern (2, 12).

Current first-line treatment for KD includes intralesional corticosteroid injections (13), topical 5-fluorouracil (14), surgical excision with intra-operative steroid injection (15) or post-operative radiotherapy (16), laser therapy (17), cryotherapy (18), and silicon occlusive dressing (15). These empirical treatments for KD remain unsatisfactory (13), with high recurrence rates of 45–100% following surgical excision without adjuvant therapy (10), underscoring the need for understanding the pathogenesis of this enigmatic condition. The prevalence of KD varies geographically, affecting 0.09% of the population in the United Kingdom, and up to 16% in the Congo (19). Interestingly, KD is extremely rare in albino individuals (20).

Inheritance patterns of KD portray an autosomal dominant trait with incomplete penetrance and variable expressivity (19). There have been reports of autosomal recessive inheritance and X-linked patterns in family case studies with multiple syndromes (21), suggesting that several genes contribute to KD predisposition (19, 22). Human leukocyte antigen polymorphisms have also been implicated in KD (23), with four single-nucleotide polymorphisms also being associated with keloid formation (24).

The pathogenesis of KD remains unknown, although there is mounting evidence implicating stem cells (25), the renin-angiotensin system (RAS) (26), vitamin D receptor (VDR) (27), and the immune system (28) involving endothelial-to-mesenchymal transition (endo-MT), a process by which endothelial cells lose their specific markers and develop a mesenchymal phenotype (29).

In this review we discuss growing evidence supporting an embryonic stem cell (ESC)-like origin of KD, and the complex interactions these primitive cells have with the RAS, hypertension, VD-related pathways and the immune system, to elucidate the pathogenesis of this challenging condition.

## Fibroblasts and Myofibroblasts in Keloid Disorder

**Figure 1:**
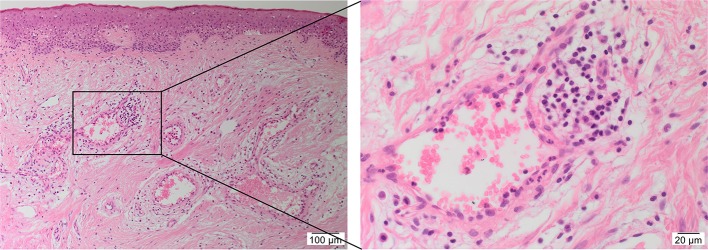
A hematoxylin and eosin stained slide of a keloid tissue sample demonstrating the presence of keloid-associated lymphoid tissues (KALTs) containing microvessels surrounded by inflammatory cells with a magnified view showing a KALT within the keloid lesion. Original magnification: 100× and 400×.

Histologically, KLs contain haphazard, thick, hyalinized eosinophilic bundles of types I and III collagen fibers with persistent immune cell infiltration sub-epidermally (30) (Figure 1). Some studies indicate hypervascularity in KLs (31), whilst others report limited microvasculature involvement associated with luminal occlusion primarily attributed to endothelial cells (32), favoring the role of hypoxia in keloid pathogenesis (33). The predominant cell types within KLs are fibroblasts and myofibroblasts, which are responsible for collagen and ECM deposition and wound contraction, respectively (34, 35). Through the regulation of the TGF-β1/Smad3 pathway, fibroblasts transition to a myofibroblast phenotype causing subsequent excessive extracellular matrix (ECM) deposition, forming a persistent pathologic scar (35, 36). However, the precise origin of these aberrant myofibroblasts remains unclear (29).

**Figure 2:**
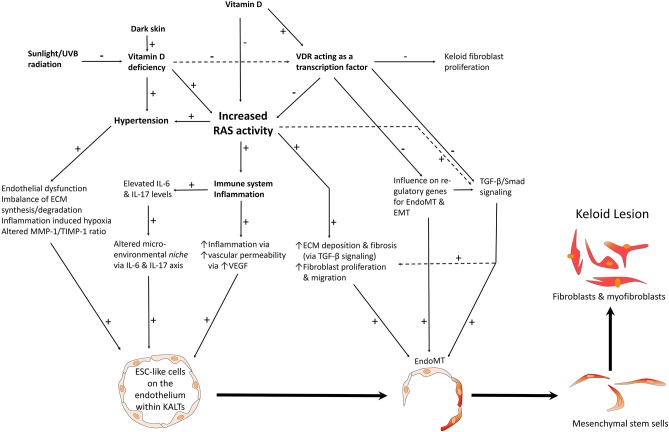
A proposed model of keloid disorder showing embryonic stem cell (ESC)-like cells within keloid-associated lymphoid tissues (KALTs), regulated by a microenvironmental *niche* with resultant proliferation and accumulation of fibroblasts and myofibroblasts in the keloid lesion (KL) via a mesenchymal stem cell intermediate through an endothelial-to-mesenchymal transition (endo-MT). The renin-angiotensin system (RAS) plays a central role in the microenvironmental *niche* with complex interactions with the immune system/inflammation, vitamin D, vitamin D deficiency (VDD), vitamin D receptor (VDR), and hypertension. VDD which is caused by reduced sunlight/UVB radiation, and leads to increased RAS activity and the resultant hypertension. VDD also directly leads to hypertension. Increased RAS activity also activates the immune system. The complex interactions between these elements lead to activation of various pro-fibrotic signaling pathways leading to generation of fibroblasts and myofibroblasts. Hypertension has a direct pro-fibrotic effect and contributes to the conducive microenvironment for the ESC-like cells within the KALTs. VDD increases RAS activity, with activation of the immune system/inflammation leading to an altered microenvironmental *niche* via the IL-6 and IL-17 axis. This increased RAS activity activates TGF-β/Smad signaling to promote EndoMT. Binding of vitamin D to VDR results in a genomic effect which counteracts the profibrotic signaling pathways. VDR transcriptional activity inhibits keloid fibroblast proliferation. VDR transcriptional activity also inhibits the pro-fibrotic TGF-β/Smad signaling pathway, down-regulates genes for EndoMT, and so may influence the formation of fibroblasts and myofibroblasts within KLs. ECM, extracellular matrix; TGF-β, transforming growth factor-β; MMP-1, matrix metalloproteinase-1; TIMP-1, tissue inhibitor of metalloproteinase-1; IL, interleukin; UVB, ultraviolet B; VEGF, vascular endothelial growth factor. “+” signifies a positive effect; “−” signifies a negative effect.

Aberrant keloid fibroblasts and myofibroblasts may be derived from an ESC-like population in keloid-associated lymphoid tissues (KALTs) (25) through a mesenchymal stem cell intermediate, by the process of endo-MT and epithelial-to-mesenchymal transition (EMT) (Figure 2), or from bone marrow mesenchymal precursors as proposed for Dupuytren's disease (29, 37, 38).

## Stem Cells in Keloid Disorder

**Figure 3:**
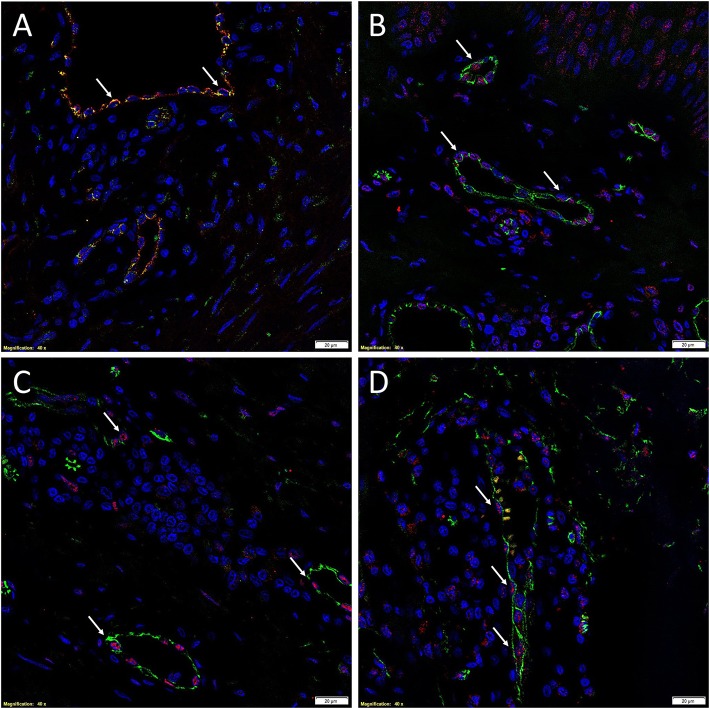
Immunofluorescence images of representative keloid tissue sections demonstrating the expression of OCT4 (**A**, green, *white arrows*) on the endothelium expressing von Willebrand factor (**A**, red). The co-expression of both proteins on the same cells was portrayed as orange **(A)**. The same endothelium, highlighted by the expression of CD34 (**B–D**, green), also expressed SOX2 (**B**, red, *white arrows*), pSTAT3 (**C**, red, *white arrows*), and NANOG (**D**, red, *white arrows*). Cell nuclei were counterstained with DAPI. Original magnification: 400X. *Reproduced with permission from the Journal of Clinical Pathology* (25).

There is increasing evidence supporting the role of stem cells in the pathogenesis of KD (28). Bagabir et al. (39) report the presence of the KALTs located within the reticular dermis, just beneath the epidermis of KLs (Figure 1). The KALTs are aggregates of inflammatory cells including T lymphocytes expressing CD3 and CD4, B lymphocytes expressing CD20, macrophages expressing CD68 and CD163, and mast cells expressing *c-kit*, tryptase, and OX40L (39). We have recently demonstrated the presence of an ESC-like population located within the endothelium of the microvessels and the perivascular tissue within the KALTs that expresses ESC markers octamer-4 (OCT4), sex determining region Y-box 2, NANOG, and phospho-signal transducer and activator of transcription 3 (Figure 3) (25). Additionally, elevated levels of OCT4 and stage specific embryonic antigen-4 have been found throughout the reticular dermis in KLs, when compared to the patient's normal skin (40). The presence of the mRNA for the ESC marker Rex-1 in KLs has also been reported (40).

Embryonic stem cells are capable of unlimited proliferation and differentiation and, with the appropriate signals, can form precursor cells of nearly all mature cell types (41). Stem cell populations previously identified, termed keloid precursor cells (KPCs), demonstrate multipotent differentiation, clonogenicity, and are proposed to be regulated by a microenvironmental *niche* conducive to keloid formation (40). We have recently demonstrated an ESC-like population within KALTs that expresses components of the RAS (26), cathepsins B, D, and G which constitute bypass loops of the RAS (42), and also VDR (27).

The ESC-like population within the KALTs that is proposed to give rise to the aberrant keloid fibroblasts and myofibroblasts expresses the RAS, its bypass loops and VDR (Figure 2).

## Renin-angiotensin System and Keloid Disorder

The RAS is an endocrine cascade integral to blood pressure, tissue perfusion, extracellular volume homeostasis, and electrolyte balance (43, 44). Renin, a rate-limiting enzyme, is released into the circulation in response to several physiological triggers (43, 44). Through cleavage by renin, angiotensinogen is converted into angiotensin I (ATI) which is subsequently hydrolyzed by angiotensin-converting enzyme (ACE) to form angiotensin II (ATII)—the primary active product of the RAS (44). Most physiological and pathophysiological roles of ATII are mediated by binding to angiotensin II receptor 1 (ATIIR_1_), causing vasoconstriction, increased blood pressure and cardiac contractility, cardiac hypertrophy, sympathetic nervous system amplification, increasing sodium retention, and angiogenesis (45, 46). Binding of ATII to ATIIR_1_ also affects cellular growth and proliferation, inflammation, oxidative stress (47), and influences immunological responses that lead to inflammatory cell recruitment and ECM deposition (48). Angiotensin II receptor 2 (ATIIR_2_) opposes the actions of ATIIR_1_, through its anti-proliferative and apoptotic functions in vascular smooth muscle, and thereby inhibiting cardiac hypertrophy and remodeling (45, 48).

The association between the RAS and tissue fibrosis is well-documented (49, 50). ATII is known to induce fibrous remodeling in several organ systems through ATIIR_1_ signaling, resulting in renal (51), cardiac (52), and idiopathic pulmonary (53) fibrosis, and silicosis and asbestosis (54). The mechanism underscoring the association between RAS activity and fibrosis is believed to be increased expression of TGF-β1 caused by ATII (55, 56), which plays a central role in fibrogenesis (55) (Figure 2). As well, over-expression of TGF-β1 has been established as a key contributor to fibrosis in nearly all tissue types, due to its stimulating effect on myofibroblast differentiation and synthesis of ECM proteins (36). It also strongly induces connective tissue growth factor, a profibrotic mediator influencing fibroblast proliferation, cellular adhesion, and ECM deposition (57) (Figure 2).

We have recently demonstrated the expression of pro-renin receptor, ACE, ATIIR_1_, ATIIR_2_ on the endothelium of the microvessels and perivascular cells within the KALTs (26). Morihara et al. (50) show significantly higher ACE activities in wounded skin and pathological scar tissue than in normal skin, implying increased levels of ATII in these conditions. Treatment with ATIIR_1_ blockers significantly reduces the pliability and vascularity of KLs and hypertrophic scars (58). This observation along with improvement of KLs following treatment with low-dose enalapril, an ACE inhibitor, further supports the role of the RAS in KD (59) (Figure 2).

**Figure 4:**
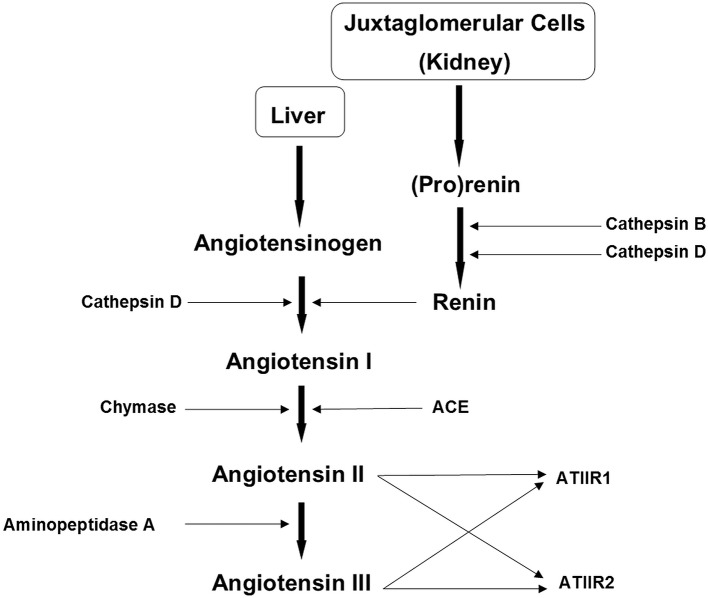
The renin-angiotensin system (RAS). In the RAS (pro)renin activation is caused by its binding to the (pro)renin receptor. Cathepsins B and D are also renin-activating enzymes. Renin and cathepsin D convert angiotensinogen to angiotensin I (ATI) which is acted upon by angiotensin converting enzyme (ACE) and chymase, to produce angiotensin II (ATII). Aminopeptidase A converts ATII to angiotensin III (ATIII). ATII and ATIII act on angiotensin II receptor 1 (ATIIR1) and angiotensin II receptor 2 (ATIIR2). Redundancy in the pathway has been revealed in the form of cathepsins B and D, aminopeptidase A and chymase. Reproduced with permission from *Integrative Cancer Science and Therapeutic* (61).

Chymase, a serine protease within mast cells, is involved in wound regeneration and pathologic scarring, and the production of ATII in tissues (60). It constitutes a bypass loop of the RAS (Figure 4) (61), having six times greater expression in KLs than in normal skin (62), and contributes to KL formation by activating TGF-β1/Smad signaling (60). It catalyzes the formation of ATII, and increases collagen I, TGF-β1, and interleukin (IL)-1β in keloid fibroblasts. Treatment of keloid fibroblasts with either the ACE inhibitor captopril or the ATIIR_1_ blocker valsartan significantly reduces mRNA expression of TGF-β1(62).

The RAS is implicated in the regulation of stem cell proliferation and differentiation, which may be the source of fibroblasts and myofibroblasts in KD (63) (Figure 2). The ESC-like population within the KALTs (39) has been proposed to be a novel therapeutic target by modulation of the RAS. Cathepsins B, D, and G are also expressed by this primitive population within the KALTs (42), and may act as bypass loops for the RAS (Figure 4) (61). This suggests the need of inhibitors of the bypass loops, in addition to classical RAS blockers, to achieve a more effective modulation of the RAS.

The pro-fibrotic nature of the RAS, which is mediated through TGF-β1 expression, promotes pathological scarring such as KD, and may regulate proliferation and differentiation of ESC-like population within the KALTs of KLs.

*In vitro*, pre-clinical and clinical studies demonstrating the effect of RAS modulating drugs on KLs and hypertrophic scars are summarized in Table 1.

**Table 1:** Studies on the effect of renin-angiotensin system modulating agents on keloid lesions and hypertrophic scars.

**References**	**Study type**	**Drug**	**Target**	**Outcome**
Hedayatvanfard et al. (58)	Pilot study	Topical losartan	ATIIR1	Reduced KL pliability and vascularity
Wang et al. (62)	*In-vitro* study	Chymostatin	Chymase	Reduced expression of angiotensin II, TGF-β, IL-1β, and COL1
Wang et al. (62)	*In-vitro* study	Captopril	ACE	Reduced expression of angiotensin II, TGF-β, IL-1β, and COL1
Enoshiri et al. (64)	Case-control study	β-blocker (carvedilol, bisoprolol, or atenolol)	Renin	Patients with either a HTS or KL were less likely to take β-blockers than those who did not develop abnormal scaring following cardiac device implantation (OR, 0.14; 95 CI, 0.01–0.87; *p* = 0.0334)
Ardekani et al. (65)	Pre-clinical study (rabbits)	Topical captopril	ACE	Decreased scar elevation index in HTS
Ardekani et al. (66)	Single case	Topical captopril	ACE	Decreased height, redness and scaling of KL with reduced pruritis
Iannello et al. (59)	Two cases	Low-dose enalapril	ACE	Near-complete resolution of KL after 15 days of treatment and complete resolution of HTS within 3–4 months of treatment in one patient. Significant improvement within 6 months of treatment in a second patient

*ACE, angiotensin-converting enzyme; ATIIR1, angiotensin II receptor 1; COL1, collagen, type 1; HSP47, heat shock protein 47; HTS, hypertrophic scar; IL-1β, interleukin-1β; KL, keloid lesion; PDGF-BB, platelet derived growth factor with two B subunits; TGF-β, transforming growth factor-β*.

## Hypertension and Keloid Disorder

Hypertension has been linked to the susceptibility for the development of KD (3), with both occurring more frequently in individuals of African descent than Caucasians (67). Patients with KD are more likely to have concomitant hypertension than patients without KD (68). Improvement of KLs in hypertensive patients has been observed following treatment with anti-hypertensive medications, such as ACE inhibitors (66) and calcium channel blockers (69). Endothelial dysfunction, which also occurs in hypertension, may also participate in the pathogenesis of KD (1, 70), and is associated with lung (71), liver (72), cardiac (73), and kidney (74) fibrosis.

In KD there is an imbalance between ECM synthesis and degradation that favors fibrosis (75). Hypertensive individuals have elevated tissue inhibitor of metalloproteinase-1 (TIMP-1), an important mediator of ECM structure (76). Interestingly, keloid fibroblasts also have elevated levels of TIMPs (75). TIMP-1 inhibits the enzyme matrix metalloproteinase-1 (MMP-1), which is responsible for collagen type 1 breakdown. Higher concentrations of TIMP-1, an inhibitor of MMP-1 seen in hypertension and keloid fibroblasts, may reduce turnover of type 1 collagen (77), which is present at higher levels in KLs (30). This hypertension-associated increase in TIMP-1 may contribute to an altered MMP-1/TIMP-1 ratio that already favors ECM increase over degradation (76) (Figure 2). Patients with hypertension treated with the ACE inhibitor lisinopril, demonstrate an increase in MMP, a decrease in TIMP-1 levels, and increased extracellular collagen maturation (78).

Hypertension is also associated with inflammation-induced hypoxia, whereby a prolonged inflammatory phase in wounding may induce interstitial hypertension, increase cellular metabolic demand within the KL causing tissue hypoxia (3, 79). This is supported by keloid fibroblasts generating ATP primarily from glycolysis, suggesting that keloid fibroblasts possess a greater capacity to survive and proliferate in a hypoxic environment (80). Evidence of reduced or occluded microvessels in the center of KLs further implicates hypoxia in KD pathogenesis (30). Furthermore, hypoxia-inducible factor-1 (HIF-1), a biomarker for local skin hypoxia, is over-expressed in KLs when compared to normal skin (81). HIF-1 activation regulates EMT, a process proposed for generating keloid fibroblasts and myofibroblasts, through the TGF-β pathway (82). A hypoxic environment has also been shown to modulate the TGF-β1/Smad3 pathway, which drives the transition of dermal fibroblasts into myofibroblasts (35).

A higher incidence of both KD and hypertension in individuals of African descent, and hypertensive individuals being more likely to develop KD than their normotensive counterparts have been observed. This suggests an interplay between these factors that may be mediated by an altered MMP-1/TIMP-1 ratio that favors ECM deposition, and hypertension-associated tissue hypoxia that increases HIF-1 expression that influences the development of aberrant keloid fibroblasts and myofibroblasts.

## Vitamin D Metabolism

In the body, vitamin D (VD) exists in two main forms: vitamin D_2_ (ergocalciferol, VD_2_) and vitamin D_3_ (cholecalciferol, VD_3_). Ultraviolet B intensity, which depends on latitude (83), and skin pigmentation determined by melanin, are key determinants of VD_3_ production (84). In this article, both VD_2_ and VD_3_ will be referred to as VD, as their respective metabolically active forms, 1,25-dihydroxyvitamin D_3_ (1,25(OH)_2_D_3_) and 1,25-dihydroxyvitamin D_2_ (1,25(OH)_2_D_2_), have comparable biologic activity upon binding to VDR (85).

Vitamin D is best known for its regulation of calcium and phosphorus homeostasis through rapid non-genomic actions by signaling intestinal calcium and phosphate absorption to maintain calcium and skeletal homeostasis (86). VDR can activate or inhibit gene expression, which can inhibit cellular proliferation, stimulate differentiation and inhibit adaptive immunity whilst promoting innate immunity (85, 87).

Vitamin D is converted to its biologically active form by hydroxylation in two steps: by 25-hydroxylation primarily in the liver to form 25-hydroxyvitamin D (25OHD) (88), which then undergoes 1α-hydroxylation by CYP27B1 to form 1,25-dihydroxyvitamin D (1,25(OH)_2_D) (85). Although the latter reaction primarily occurs within the proximal renal tubule, this enzyme is also found in several extra-renal sites, including T and B lymphocytes (89, 90). Given that KALTs consist of lymphoid aggregates (39), CYP27B1 may be present in the KALTs.

Vitamin D exerts its biologic effects through genomic and non-genomic actions. It binds to VDR, a DNA-binding transcription factor from the superfamily of steroid hormone nuclear receptors (89) (Figure 2). When VD binds to VDR, an active signal transduction complex consisting of VD liganded to VDR and the retinoid X (RXR) is formed. The VDR-RXR heterodimer is able to recognize VDREs on VD regulated genes and to have genomic effects (91), with thousands of VDREs, and hundreds of genes containing VDREs, having been identified.

CYP27B1 may be expressed by lymphocytes within KALTs leading to local production of VD which may then exert genomic effects via VDR signaling on ESC-like cells expressing VDR within KALTs. Investigation into the presence of CYP27B1 in lymphocytes within the KALTs warrants investigation.

## Vitamin D Deficiency in Keloid Disorder

Individuals with KD are more likely to develop VD deficiency (VDD) (92), hypertension (3), and be dark-skinned (67). Both VDD and hypertension are also more prevalent in dark-skinned populations (67, 92).

The primary source of VD comes from sunlight exposure (88), with skin pigmentation being the key determinant in reduced UVB penetration, leading to decreased cutaneous synthesis of VD_3_(83) (Figure 2). Very dark-skinned individuals, as in some African populations, may have a sun protection factor of up to 15, which absorbs up to 99% of UVB radiation and consequently decreases VD_3_ synthesis by up to 99%, thus increasing the susceptibility to VDD (93, 94).

Keloid fibroblasts demonstrate reduced proliferation in response to VD_3_ treatment in a dose-dependent manner, with collagen I expression decreasing three-fold in the treated samples, supporting the influence of VD_3_ on keloid regression (95). Following this treatment, the gene expression pattern of the anti-apoptotic factor Bcl-2 reduces significantly over time, and after 24 h, the level of pro-apoptotic caspase-3 increases five-fold (95) (Figure 2). The effect of VD on Bcl-2 and caspase-3 is relevant since keloid fibroblasts have increased resistance to apoptosis compared with normal skin fibroblasts (96).

Vitamin D receptor acts as a negative regulator of TGF-β1/Smad signaling (Figure 2). VDR knockdown enhances fibroblast sensitivity to TGF-β1 in patients with systemic sclerosis, and activation of VDR with paricalcitol reduces the stimulatory effect of TGF-β1 on fibroblasts, and so inhibits collagen production and myofibroblast differentiation. Furthermore, paricalcitol increases formation of VDR and phosphorylated Smad3 complexes, inhibiting Smad transcriptonal activity (97), which regulates the expression of various profibrotic genes, including MMPs, various proteoglycans, integrins, and plasminogen activator (98). The genes for plasminogen activator and VDR have been identified as susceptibility genes for KD (92).

Expression of VDR is significantly lower in the peripheral blood lymphocytes of individuals with KD (92), in the epidermis of KLs (99), and in fibroblasts of patients with systemic sclerosis (97), another fibrotic condition. We have previously demonstrated that the ESC-like population within the KALTs also expresses VDR (27), in addition to components of the RAS (26), and cathepsins B, D, and G which constitute bypass loops of the RAS (42).

Upon binding to VDR, the genomic effects of VD may regulate expression of genes regulating EMT (100), endo-MT and subsequent cellular differentiation and proliferation, and hence may contribute to the formation of keloid fibroblasts and myofibroblasts via a mesenchymal stem cell intermediate, in KD (28) (Figure 2). Further work is needed to elucidate whether VDD influences the proliferation and differentiation of ESC-like cells within the KALTs through genomic actions of VD.

The observation that darker skinned individuals are predisposed to VDD, hypertension and KD, may give clues to the pathogenesis of KD. The anti-proliferative effect of VD through reduced Bcl-2 and increased caspase-3 expression, its inhibition of TGF-β1/Smad signaling, and its potential influence on the ESC-like cells within KALTs, underscore the need of investigation that may lead to potential therapy of KD with VD supplements.

## Vitamin D Deficiency Increases Renin-angiotensin System Activity

Vitamin D deficiency increases RAS activity which exacerbates fibroproliferative conditions such as pulmonary fibrosis (49); this may also occur in KD. Moreover, inhibition of the RAS by VDR activation has been shown *in vitro* (101) and *in vivo* (102), suggesting that VD acts as an endogenous RAS inhibitor (103) (Figure 2). Through increased expression of TGF-β and its stimulatory effect on myofibroblast differentiation and ECM deposition, elevated RAS activity ultimately induces tissue fibrosis via the TGF-β signaling pathway (36, 57) (Figure 2). Additionally, VDR interacts with Smad3 to form a VDR-Smad3 complex (104), further indicating that VD_3_ interacts with the TGF-β signaling pathway via VDR. The complex resulting from interaction of TGF-β with Smad, translocates to the nucleus to regulate transcription of genes in the TGF-β family, which regulate ECM (105).

Chun et al. (102) show that VD_3_ negatively and directly regulates renin gene transcription, via a VDR-mediated process (Figure 2), and that VDR-null mice have elevated plasma ATII production, are hypertensive and have abnormal drinking behavior. The VDR-mediated suppression of renin is confirmed by the observation that treatment of wild-type mice with VD_3_ over three days suppresses renin expression by 50%. They also report that wild-type mice with reduced VD_3_ biosynthesis display elevated expression of renin, which is reversed by injection of VD_3_, and that VD_3_ directly suppresses transcription of the renin gene in transfected As4.1 cells via a VDR-dependent mechanism. However, it has also been reported that VD given to hypertensive animals reduces blood pressure independent of renin levels (106).

Increased RAS activity by a VDR-mediated effect leads to increased renin levels, resulting in increased plasma ATII levels which cause hypertension in mice. If these same observations occur in humans, hypertension resulting from a VDR-mediated effect may impact on the ESC-like cells within the KALTs (25) that express the RAS (26) and its bypass loops (42) (Figure 2).

## Microenvironmental *Niche* and Vitamin D in Keloid Disorders

Stem cells that possess self-renewal and pluripotent capabilities, are regulated by both intrinsic genetic programs and extrinsic signals (107). The former involve genes, including those expressed on ESC-like cells within the KALTs (25), and the latter arising from the microenvironmental *niche* (40), which includes all immediate ECM constituents and the surrounding cells (40, 108). For ESC-like cells within the KALTs to be affected by the microenvironmental *niche*, intrinsic programs respond to changes in the surrounding *niche* (107). In normal physiologic conditions, a microenvironmental *niche* prevents aberrant stem cell proliferation by providing the balance between proliferation-inhibiting and proliferation-promoting signals (40, 109). ESC-like cells within the KALTs (25) and tumor-like cells expressing ESC markers in the reticular dermis (40), may in fact be the same cell populations. Dysregulation of this microenvironmental *niche* may cause uncontrolled proliferation, self-renewal (110), and differentiation (37) of the ESC-like cells via endo-MT, giving rise to the fibroblasts and myofibroblasts within KLs (28) (Figure 2).

Inflammation is initiated in response to injury or infection (111)—a protective process shown to regulate stem cell *niches* (112). There is mounting evidence demonstrating innate and adaptive immune systems being central to wound repair and tissue regeneration (113), during which, released cytokines and growth factors contribute to the excessive cellular proliferation and ECM deposition in KD (30). An increase of the inflammatory cytokines IL-6 and IL-17 plays a critical role in the microenvironmental *niche* within KLs by regulating self-renewal and differentiation capability of KPCs (40), and possibly, the ESC-like cells within the KALTs (25) (Figure 2). IL-6 is an influential regulator of stem cell differentiation and self-renewal (114, 115), and is implicated in the development of most fibrotic conditions (116), with IL-17 being implicated in some inflammatory conditions (117, 118). The levels of both IL-6 and IL-17, and their respective receptors, are elevated within KLs, and are implicated in the pathogenesis of KD (40, 119). Other cytokines that are elevated within KLs include ATII, TGF-β1, vascular endothelial growth factor (VEGF) (Figure 2), oncogene-α, IL-1β, TNF-α, RANTES, platelet-derived growth factor-β homodimer, and stem cell factor (40).

Zhang et al. (40) have demonstrated that IL-6 increases expression of OCT4 and telomerase, which are regulators of stem cell function, *in vivo*, supporting the role of IL-6 in KD. IL-6 has been shown to promote growth of transplants from KPCs *in vivo* which show histological features characteristic of KLs. Moreover, administration of IL-6 neutralizing antibodies decreases telomerase expression and the growth of KPC-generated transplants. IL-6 functions both upstream and downstream of IL-17, creating a positive feedback loop that promotes autoimmunity (120). IL-6 and TGF-β1, which are elevated in KLs, promote differentiation of T_H_17 cells which then produce IL-17, further enhancing IL-6 release from keloid stem cells to influence the microenvironmental *niche* (40). This IL-6/IL-17 positive feedback loop is one factor contributing to the altered inflammatory microenvironmental *niche*, that may possibly control the proliferation and transdifferentiation of ESC-like cells within the KALTs (Figure 2).

The altered microenvironmental *niche* within KLs regulated by positive feedback loops involving IL-6 and IL-17 and other cytokines, may promote the stemness and proliferation of the ESC-like within the KALTs within KLs.

## Immune System and Vitamin D in Keloid Disorders

Bagabir et al. (39) show that the number of CD3^+^ and CD4^+^ T lymphocytes underneath the epidermis overlying KLs is significantly higher than in normal skin and scar tissue (121). VDR expressed on CD4^+^ cells has been shown to have high affinity for VD (122), and T_H_17 cells which produce IL-17 have many VDR transcripts (123). VD is known to influence the immune system by targeting CD4^+^ cells (123), as demonstrated by data showing VDR expression on hematopoietic cells is required for inhibition of experimental autoimmune encephalomyelitis in chimeric mice (123). This research concludes that VD has autoimmune-protective effects via VDR signaling in CD4^+^ T lymphocytes (123, 124), which are present at higher levels in KLs than in normal skin and scar tissue (39).

It is interesting that VD affects cells that are in higher proportions in KLs than in normal skin, and that abundant VDR transcripts are found within IL-17 producing T_H_17 cells that may contribute to the IL-6/IL-17 axis that may regulate primitive cells in KLs (Figure 2). Furthermore, the fact that KD has autoimmune characteristics that may be mediated by autoimmune responses (125), indicates that immunological effects caused by VD may impact upon the immune response seen in KD, ultimately affecting the keloid microenvironmental *niche*, and thus potentially the ESC-like cells on KALTs.

The RAS which is expressed on ESC-like cells within the KALTs is implicated in stem cell proliferation and differentiation (63, 126), and also has a range of effects on both innate and adapative immunity (126) with inflammatory cells possessing all the components of the RAS (127). Activation of the RAS has strong pro-inflammatory effects through increasing vascular permeability, expression of inflammatory cytokines, chemokines, induction of reactive oxygen species (ROS), as well as other processes, primarily through ATIIR_1_ activation (128). Increased expression of the aforementioned pro-inflammatory molecules by ATIIR_1_ binding occurs through activation of various pathways in which there is stimulation of the NF-kB pathway, which can be attenuated by VD to produce an anti-fibrotic effect (129). Moreover, ATIIR_1_ activation stimulates fibroblast proliferation and collagen synthesis (130). Lastly, ATII binds to the tranduction protein signal pSTAT3, an ESC marker expressed by ESC-like cells within the KALTs (25), to mediate inflammation, cell proliferation and differentiation (131).

Vitamin D may produce auto-immune protective effects that impact on the microenvironmental *niche* within KLs to alter the behavior of the ESC-like population within the KALTs. Activation of the NF-kB pathway, its limited attenuation in VDD, and increased fibroblast proliferation and ECM production caused by RAS activation, are hallmarks of pathological scarring characteristic of KD (Figure 2).

## Conclusion

Despite intensive research, treatment for KD remains empirical and unsatisfactory. Mounting evidence suggests a central role for an ESC-like population within KALTs. These cells can be regulated by an microenvironmental *niche* centered on the RAS including its bypass loops consisting of enzymes such as cathepsins B, D, and G, and on converging signaling pathways including VDR-mediated mechanisms and the immune system, resulting in proliferation and accumulation of keloid fibroblasts and myofibroblasts. Further elucidation of the complex interaction between the RAS, VDR, other compounds, and the immune system that collectively form the microenvironmental *niche* may lead to novel targeted therapies for this enigmatic and challenging condition.

## Author Contributions

EK and ST drafted the manuscript. EK, CP, PD, HB, and ST critically revised the manuscript. HB provided the H&E images. All authors commented on and approved the manuscript.

### Conflict of Interest

ST and PD are inventors of a PCT application Treatment of Fibrotic Conditions (PCT/NZ2016/050187). The remaining authors declare that the research was conducted in the absence of any commercial or financial relationships that could be construed as a potential conflict of interest.
